# Differential Protein Expression among Two Different Ovine ARDS Phenotypes—A Preclinical Randomized Study

**DOI:** 10.3390/metabo12070655

**Published:** 2022-07-15

**Authors:** Karin Wildi, Mahe Bouquet, Carmen Ainola, Samantha Livingstone, Sebastiano Maria Colombo, Silver Heinsar, Noriko Sato, Kei Sato, Emily Wilson, Gabriella Abbate, Margaret R. Passmore, Kieran Hyslop, Keibun Liu, Gianluigi Li Bassi, Jacky Y. Suen, John F. Fraser

**Affiliations:** 1 Critical Care Research Group, The Prince Charles Hospital, Brisbane 4032, Australia; m.bouquet@uq.edu.au (M.B.); carmen.ainola@gmail.com (C.A.); samantha.livingstone@uqconnect.edu.au (S.L.); sebastiano.colombo@gmail.com (S.M.C.); silverheinsar@gmail.com (S.H.); laugh10948@gmail.com (N.S.); m02045ks@gmail.com (K.S.); emily.wilson@uq.edu.au (E.W.); g.abbate@uq.edu.au (G.A.); m.passmore@uq.edu.au (M.R.P.); k.hyslop@uq.edu.au (K.H.); keibun.liu@uq.edu.au (K.L.); g.libassi@uq.edu.au (G.L.B.); j.suen1@uq.edu.au (J.Y.S.); fraserjohn001@gmail.com (J.F.F.); 2 Medical Faculty, The University of Queensland, St. Lucia, Brisbane 4067, Australia; 3 Department of Cardiology, Cardiovascular Research Institute Basel, University Hospital Basel, University of Basel, 4031 Basel, Switzerland; 4 Department of Anaesthesia and Intensive Care Medicine, Fondazione IRCCS Ca’ Granda Ospedale Maggiore Policlinico, 20122 Milan, Italy; 5 Medical Faculty, Queensland University of Technology, Brisbane 4059, Australia; 6 Uniting Care Hospitals, St Andrews War Memorial and The Wesley Intensive Care Units, Brisbane 4001, Australia

**Keywords:** protein expression profiles, SWATH, Acute Respiratory Distress Syndrome (ARDS), phenotypes, ovine model

## Abstract

Despite decades of comprehensive research, Acute Respiratory Distress Syndrome (ARDS) remains a disease with high mortality and morbidity worldwide. The discovery of inflammatory subphenotypes in human ARDS provides a new approach to study the disease. In two different ovine ARDS lung injury models, one induced by additional endotoxin infusion (phenotype 2), mimicking some key features as described in the human hyperinflammatory group, we aim to describe protein expression among the two different ovine models. Nine animals on mechanical ventilation were included in this study and were randomized into (a) phenotype 1, *n* = 5 (Ph1) and (b) phenotype 2, *n* = 4 (Ph2). Plasma was collected at baseline, 2, 6, 12, and 24 h. After protein extraction, data-independent SWATH-MS was applied to inspect protein abundance at baseline, 2, 6, 12, and 24 h. Cluster analysis revealed protein patterns emerging over the study observation time, more pronounced by the factor of time than different injury models of ARDS. A protein signature consisting of 33 proteins differentiated among Ph1/2 with high diagnostic accuracy. Applying network analysis, proteins involved in the inflammatory and defense response, complement and coagulation cascade, oxygen binding, and regulation of lipid metabolism were activated over time. Five proteins, namely LUM, CA2, KNG1, AGT, and IGJ, were more expressed in Ph2.

## 1. Introduction

More than five decades since its first description in 1967 [1], Acute Respiratory Distress Syndrome (ARDS) remains a life-threatening critical illness that affects 10% of intensive care unit patients on mechanical ventilation, with a mortality rate of 45% in the most severe cases [2,3]. ARDS is implicated in up to 85% of all COVID-19 deaths—now estimated as the fourth leading cause of death globally since the start of 2020 [4]. Until recently, there have been minimal advances in the understanding of the pathophysiology of ARDS [5,6]. The syndromic nature of ARDS, its broad etiological heterogeneity, as well as its variable host responses have impeded significant progress in comprehensive research, resulting in a consistently high mortality [2] and morbidity rate [7] worldwide.

Recent post-hoc analyses of large clinical ARDS trials within the National Health Lung and Blood Institute (NHLBI) network revealed evidence for distinct ARDS subgroups, defined by specific clinical and biological features: a hypoinflammatory (P1) and a hyperinflammatory subphenotype (P2) [8,9,10,11,12,13]. In brief, P2 is characterized by a more severe shock and inflammatory state, hemodynamic alterations, and non-pulmonary organ failure, as well as significantly higher mortality [8,9,10,11,12,13]. Additionally, retrospective analyses of overall neutral or negative ARDS treatment studies demonstrated a possible interaction between the ARDS subphenotype and the benefit of a specific management strategy [8,10] or anti-inflammatory treatment [11,14]. Therefore, early identification of ARDS subphenotypes [13,15,16] combined with targeted treatment might offer a new therapeutic approach [17,18].

Whilst promising, the current data based on subphenotypes are all retrospective in nature, and, most importantly, at this time, little is known about the biological processes over time driving the disease, and there is no tool set available for the early identification of the respective subphenotype at bedside. There have been several efforts in the quest for biomarkers in ARDS in the past [19,20,21], some using a genomics and/or proteomics approach [22]. As proteins represent a dynamic expression of cell function, proteomics is likely a more powerful tool in ARDS for biomarker discovery and the understanding of biological pathways than genomics. In subphenotypes, this process has only recently started [9,23]. Because of the syndromal nature of ARDS, and its complexity, multifactorial causes, and risk factors, the discovery of a single biomarker remains unlikely. However, subphenotyping based on protein patterns might help in narrowing the field through predictive enrichment [15,24] and ultimately lead the approach towards targeted medicine in ARDS [25,26].

Our group previously assessed different models of lung injury in an ovine model, and demonstrated that one specific ARDS induction method mimics the key features observed in the human ARDS P2 subphenotypes [27]. Given that a) inflammatory pathways in ovine models and humans are comparable in many aspects [28] and that b) results from a recent analysis showed similar gene expression patterns in human P2 and lipopolysaccharide (LPS)-induced ARDS (as used in our Ph2 ovine model) in animal models [23], we hypothesize that our ARDS models offer the opportunity to study protein expression among different ARDS subgroups more closely.

We therefore aim to describe quantitative protein abundance among two different ovine ARDS models: phenotype 1 (Ph1) and phenotype 2 (Ph2).

## 2. Results

### 2.1. Studied Population

Baseline characteristics of studied animals did not differ among Ph1 and Ph2 and are shown in Table S1 in the Supplementary Materials. After induction of ARDS, Ph2 animals showed more metabolic disturbances and hemodynamic alterations. Selected clinical and laboratory parameters during the experiment (at T12 and T24) are shown in Table S2 (Supplementary Materials).

Inflammatory cytokines (interleukin-6 (IL), -8, -10) displayed an early peak in Ph2 around 2 h. Furthermore, while IL-8 and IL-10 then assimilated to Ph1 levels over the observation period, IL-6 remained elevated in Ph2 animals (Figure S1, Supplementary Materials).

The Lung Injury Score (LIS) was 0.32 (0.28–0.37) among Ph1 and 0.35 (0.30–0.41) among Ph2 animals (Figure S2, Supplementary Materials).

### 2.2. Unsupervised Cluster Analysis

**Figure 1:**
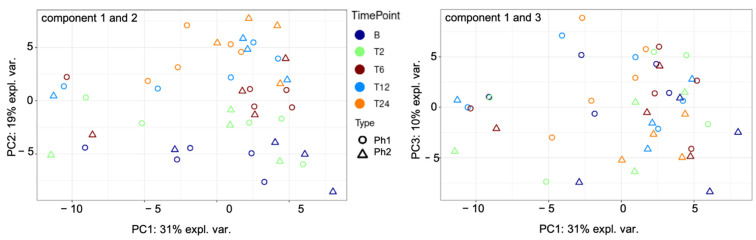
PCA components 1 to 3 among Ph1 and Ph2 for all time points. Abbreviations: B: baseline, T: time point; Ph1/2: phenotypes 1 and 2; expl. var.: explained variability; PC: principal component.

Principal component analysis (PCA) was performed to visualize differences and similarities among Ph1 and Ph2. It resulted in distinctive patterns, with PC1 (principal component) explaining 31%, PC2 19%, and PC3 10% of the variability among samples. Figure 1 displays the discovered clusters among phenotypes and sampling time points.

Correlation of all samples is shown in Figure S3 (Supplementary Materials).

### 2.3. Proteins of Differential Abundance

After quality control and log-normalization, 198 proteins remained for analysis. In total, 45 proteins were detectable in every sample, 91 proteins in >75%, 120 in >50%, and 160 proteins in >25% of samples.

**Figure 2:**
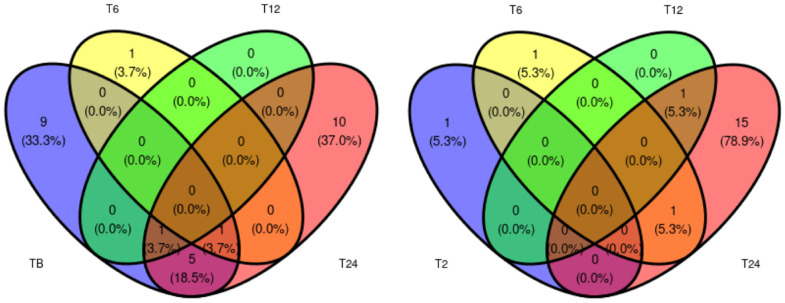
Venn diagram displaying number of differentially expressed proteins between Ph1 and Ph2 at assessed time points. Abbreviations: B: baseline, T: time point.

Differential abundance between Ph2 and Ph1 at each time point is shown Figure 2. A comprehensive table of all differentially expressed proteins between Ph1 and Ph2 at every time point can be found in Table S3 (Supplementary Materials). Overall, many uniquely expressed proteins among Ph1 and Ph2 were detected, but little shared expression between time points.

### 2.4. Supervised Cluster Analysis

**Figure 3:**
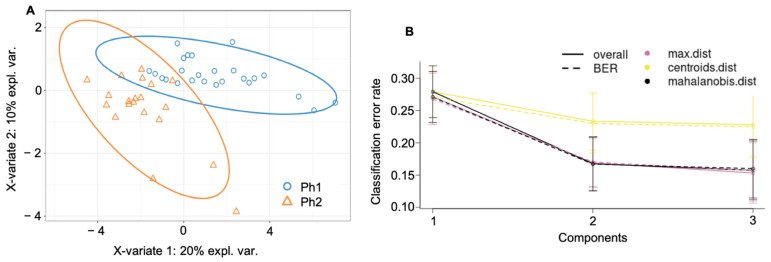
Cluster analysis among both phenotypes and all time (**A**) final PL-SDA (**B**) error rate of the final PLS-DA. Abbreviations: Ph1/2: phenotypes 1 and 2; expl. var.: explained variability; BER: balanced error rate; max. dist.: maximal distance; centroids dist.: centroids distance.

In the partial least squares discriminant analysis (PLS-DA), the best number of components was determined to be three and the optimal number of proteins to keep per component was manually selected as 20, 3, and 10 according to Figure S4 (Supplementary Materials). This resulted in two distinctive protein signatures among Ph1 and Ph2, as shown in Figure 3A, with a final error rate per component of 0.15 to 0.27 (Figure 3B).

The final class error rate among phenotypes as expressed in terms of maximal distance (max. dist.) was generally low; for Ph1, it resulted in 29.7% (component 1; C1), 15.8% (component 2; C2), and 12.3% (component 3; C3), and for Ph2, in 23.9% (C1), 18.3% (C2), and 19.1% (C3).

The PLS-DA components resulted in an AUC of 0.916 (C1), 0.992 (C2), and 1.0 (C3) (all *p* < 0.0001) for distinction between Ph1 and Ph2. Individual loadings of components 1 to 3 are reported in Table 1.

### 2.5. Analysis of Specific Proteins among Phenotypes over Time

We report on the top 34 proteins with a relaxed *p* to <0.1 to allow reasonable pathway analysis.

**Figure 4:**
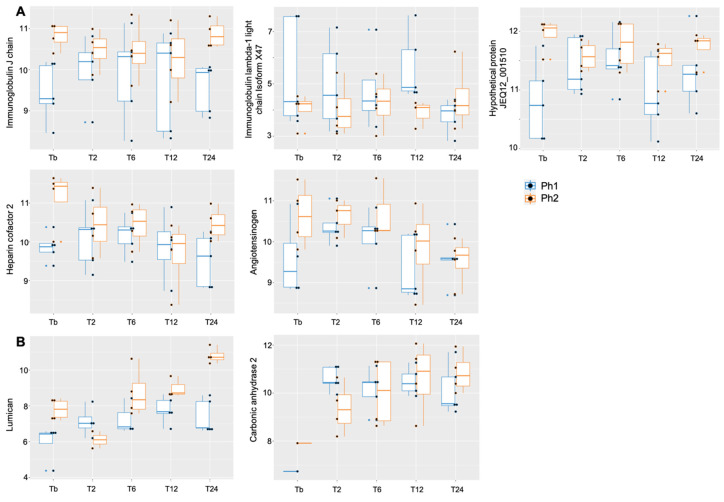
Boxplot for proteins with relaxed *p* < 0.1 (**A**) among Ph1/2 and (**B**) for interaction phenotype:time, *X*-axis displaying time points, *y*-axis displaying log2 value of respective protein. Abbrev: Tb: baseline, T: time point; Ph1/2: phenotypes 1 and 2.

The linear mixed-effect models (LMM) showed a difference between Ph1 and Ph2 for immunoglobulin J chain, hypothetical protein JEQ12_001510, heparin cofactor 2, angiotensinogen (upregulated in Ph2), and immunoglobulin lambda-1 light chain isoform X47 (downregulated in Ph2) (Table 2A, Figure 4A). A significant interaction effect for phenotype:time was seen in lumican and carbonic anhydrase 2 (Table 2C, Figure 4B). Over the observation time of the study, 27 proteins showed a significant up- or downwards trend (Table 2B).

### 2.6. Pathway Analysis

Identification of all “JEQ hypothetical proteins” among the top 33 identified ones in LMM (*p* < 0.1 among Ph1/2, over time and/or for phenotype:time interaction) was done with a peptide BLAST (at least 99% percent identity required; Table 3) in order to maximize the input for pathway analysis. Then, a STRING analysis was conducted on all proteins identified as potentially relevant with LMM.

The input consisted of 33 proteins, resulting in 22 nodes and 49 edges, with an average node degree of 4.45 (11 proteins could not be identified by STRING). The average local clustering coefficient was 0.591 and the protein–protein interaction (PPI) enrichment *p*-value was determined to be <0.0001.

**Figure 5:**
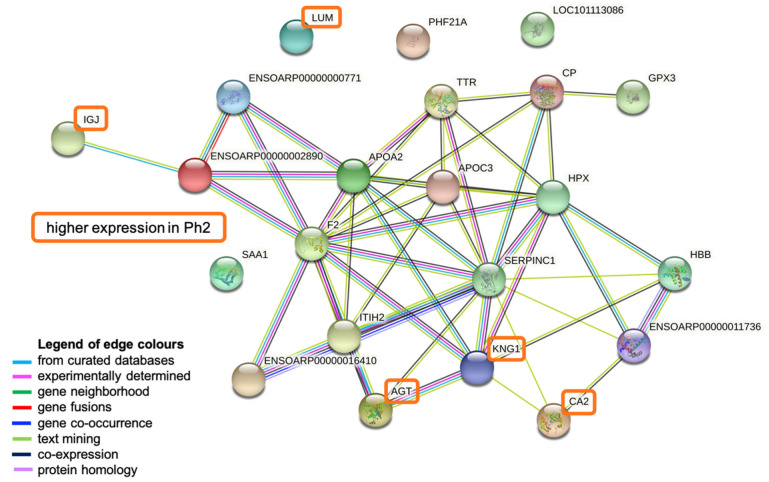
STRING network analysis. Figure created in STRING Database (STRING Consortium 2022, www.string-db.org). Abbreviations: LOC101113086: primary amine oxidase, lung isozyme; APOA2: apolipoprotein A-II; HPX: hemopexin; CP: ceruloplasmin precursor; ENSOARP00000000771: uncharacterized protein; ITIH2: inter-alpha-trypsin inhibitor heavy chain H2; HBB: hemoglobin subunit beta; LUM: lumican; IGJ: immunoglobulin J chain; ENSOARP00000011736: hemoglobin subunit alpha; CA2: carbonic anhydrase 2; AGT: angiotensinogen; SAA1: serum amyloid A protein; ENSOARP00000016410: uncharacterized protein, belongs to serpin family; TTR: transthyretin; PHF21A: PHD finger protein 21A; APOC3: apolipoprotein CIII; F2: thrombin; ENSOARP00000002890: complement C4-like isoform X1; GPX3: glutathione peroxidase 3; SERPINC1: antithrombin-III precursor; KNG1: kininogen-1 isoform X2.

The most dominant biological process found to be activated was the complement and coagulation cascade (False Discovery Rate (FDR) < 0.001%) displaying antithrombin-III precursor (SERPINC1), thrombin (F2), kininogen-1 isoform X2 (KNG1), inter-alpha-trypsin inhibitor heavy chain H2 (ITHI2), complement C4-like isoform X1 (ENSOARP00000002890), and ENSOARP00000000771 (uncharacterized protein). The same proteins except F2, but with the addition of apolipoprotein CIII (APOC3), apolipoprotein A-II (APOA2), and a serpin family protein (ENSOARP00000016410), are involved in the negative regulation of catalytic activity (FDR 0.0015%). Oxygen binding and carrier activity (FDR both <0.03%) was represented by hemoglobin subunit alpha (ENSOARP00000011736) and hemoglobin subunit beta (HBB). Other dominant biological processes include the inflammatory (FDR 0.023%; KNG1, F2, serum amyloid A protein (SAA1), ENSOARP00000002890, ENSOARP00000000771) and the defense response (FDR 0.031%; same proteins; additionally, Immunoglobulin J (IGJ), APOA2). APOA2 and APOC3 participate in the (negative) regulation of cholesterol and lipid metabolism (FDR 0.012%) (Figure 5, Table S4).

## 3. Discussion

This is a comprehensive animal model of ovine ARDS phenotypes, in which the novelty is an observation time of up to 24 h and targeted sampling for protein assessment to discover a protein signature for differentiation among the two phenotypes.

The discovery of ARDS subphenotypes in the heterogenous critical care population is a promising new approach to decreasing ARDS mortality with phenotype-specific treatment, yet it is supported only by retrospectively collated evidence and the underlying biological processes are poorly understood. In addition, a biomarker signature that can easily and reliably differentiate among the subphenotypes at bedside is still missing. The similarities of the ovine and human inflammatory pathways and the possibility to induce an ovine lung injury, mimicking the features of the human hyperinflammatory ARDS subphenotype, gave us the opportunity to develop a highly controlled study to further explore the distinct biological processes in ovine ARDS.

We report five main findings: firstly, in the unsupervised cluster analysis, there were patterns in proteins emerging over the study observation time, resulting from time-specific biological processes. Second, protein patterns were slightly more pronounced by the factor of time than different injury models of ARDS. Third, among the 198 proteins analyzed using supervised clustering, it was apparent that a signature consisting of 33 proteins was able to differentiate best among Ph1/2 with a low error rate and good diagnostic accuracy. Fourth, network analysis showed that specific proteins involved in the inflammatory and defense response, complement and coagulation cascade, oxygen binding, and regulation of lipid metabolism were activated over time. Fifth, five proteins, LUM, CA2, AGT, KNG1, and IGJ, were more expressed in Ph2.

The finding that protein patterns among time points were more pronounced than among ARDS phenotypes is relatively surprising, as our previous results clearly show that the two ovine phenotypes are different in their biological responses and cytokine profiles [27]. The validity of our Ph2 model has been highlighted by a recent preprint [23] that showed that gene expression in animal LPS models is comparable to that of the human hyperinflammatory ARDS subphenotype; therefore, similar underlying molecular pathways are potentially activated.

The STRING interaction enrichment parameters indicate that the input proteins in the pathway analysis show interactions that are clearly not random but instead at least partially biologically connected. The most dominant active biological processes in both ovine ARDS phenotypes were the complement and coagulation cascade, oxygen binding and carrier activity, negative regulation of catalytic activity, inflammatory and defense response, as well as the regulation of cholesterol and lipid metabolism. These findings are in line with previously identified key biological processes in the disease: in particular, the defense response, inflammation, and coagulation processes are largely intertwined [29] in a presumably inflammatory disease. In a syndrome where disturbances in gas exchange are the most important characterizing feature, oxygen binding and carrier activity is hardly surprising as an involved biological process [30]. The involvement of proteins of the cholesterol and lipid metabolism in ARDS has been shown previously [31,32] as active regulators of host immune responses by inhibiting the expression of adhesion molecules. APOC2 and APOC3 are also able to bind and consecutively neutralize LPS and endotoxins [32]; however, there was no overexpression detectable in LPS-induced Ph2. A possible explanation is that proteins of lipid metabolism have been detected in mainly direct (epithelial) lung injury in human ARDS [31], while both of our phenotypes received OA, which is known to cause a direct injury to the pulmonary epithelium [33].

Six proteins were clearly more expressed in Ph2 than Ph1, in the LMM and in the graphical display; five could be identified in the pathway analysis. LUM belongs to the proteoglycan family and is involved in collagen fibrillogenesis within the extracellular matrix [34]. In an analysis of alveolar macrophages in sepsis-induced ARDS, among the 10 proteins that were upregulated early in the course of ARDS [35], organizing proteins of the cytoskeleton were involved as well. CA2 and AGT are both part of the angiotensin-activated signaling pathway, thereby involved in hemodynamic processes by the regulation of arterial blood pressure [36]. Additionally, CA2 is a regulator of homeostatic processes through the dipeptide transmembrane transport, therefore heavily involved in metabolic compensation processes. Ph2 animals expressed a higher heart rate during the first few hours and a higher cardiac index throughout the experiment, pointing towards an increased need for the regulation of hemodynamic processes. Additionally, Ph2 animals had a more negative base excess with a higher lactate, indicating a more unstable metabolic condition and therefore potentially resulting in upregulated activity of CA2 for metabolic compensation. IGJ links two monomer units of IgM or IgA, and is therefore part of the humoral and adaptive immune response [37]. The higher levels of IGJ in Ph2 may indicate that the humoral immune response is more pronounced in Ph2 animals. KNG1 is active as two proteins: high-molecular-weight kininogen (HMWK) and low-molecular-weight kininogen (LMWK). Both play an important role in coagulation processes by the inhibition of platelet aggregation [38]. HMWK participates in inflammatory processes through the release of bradykinin and consecutively prostaglandins; additionally, it has a direct effect on the vascular contraction state and permeability. All processes are likely more pronounced in our Ph2 ARDS model.

Important features of ARDS were first described in animal models, well before its first official description in humans by Ashbaugh in 1967 [39,40]. Due to important similarities in pathophysiology regarding pulmonary and circulatory mechanics, as well as inflammatory pathways, large animal models have helped us to understand and apply now widely used key concepts in ARDS management: ventilator-induced lung injury [41,42] and prone positioning [43,44]. Our animal model of ARDS phenotypes, with the Ph2 expressing similar traits to the human hyperinflammatory subphenotype [23], offers an opportunity to study biological patterns that are potentially translatable to the human ARDS population. The next steps in the translation of our results would be (a) to repeat the analysis in more biological replicates of Ph2 and Ph1 to detect proteins that were potentially masked by high abundance proteins, (b) to compare protein patterns between plasma and bronchoalveolar fluid as originating from the location of the injury, and (c) to compare expression profiles among ovine models to human expression patterns in P1 and P2 to reveal the true extent of translatability among large animal models and the human ARDS population. There is a clear trend towards personalized medicine in syndromal diseases in critical care medicine [25,26,43]. Large animal models sharing key traits with human subphenotypes might offer a step along the way to a better understanding of specific subgroups and ultimately towards personalized medicine.

Strengths of this study include the use of an established and reliable model of ovine ARDS phenotypes [23,27], a controlled setting with a defined injury time point, sampling over the study observation time, and a randomized animal allocation.

We report several limitations. Firstly, we did not perform albumin depletion; therefore, the discovery rate of low-abundance proteins may be lower than expected. However, the total amount of identified proteins is in line with previous reports [31,45,46,47]. Second, the sampling time was limited to up to 24 h as we were interested in early differentiation among phenotypes. We can only hypothesize that there might be more differentially expressed patterns further down the track. Third, with five and four, respectively, the number of biological replicates was limited; additionally, we did not have control animals for analysis due to ethical reasons. Fourth, we did not take blood cultures; therefore, we cannot know if these animals developed infections over the course of the study that potentially influenced the results. Fifth, the known limitations of non-targeted proteomics are to be noted [22]. Sixth, both injury models received OA; therefore, we do not know what would be the protein expression of OA as compared to LPS alone.

## 4. Materials and Methods

Animal studies were conducted at the Queensland University of Technology (QUT) Medical Engineering Facility (MERF) in Brisbane. Animal ethics was approved by the QUT Office of Research Ethics and Integrity (No. 18-606).

ARDS was induced according to the definition and guidance of Acute Lung Injury in experimental animal models as provided by the American Thoracic Society (ATS) [48,49].

### 4.1. Animal Model

Animal experiments were approved by the QUT Office of Research Ethics and Integrity (No. 18-606), in accordance with the Australian Code of Practice for the Care and Use of Animals for Scientific Purposes and the Animal care and Protection Act 2001 (QLD).

This study is a secondary analysis of control animals of Ph1 and Ph2 ARDS from a blinded, randomized, controlled preclinical trial in an ovine model. A total of 9 female non-pregnant Merino-Dorset crossbreed ewes, aged 1–3 years, mean weight 50 ± 5 kg, were included in this analysis and randomly assigned to one of the two groups: Ph1 (*n* = 5) and Ph2 (*n* = 4). Randomization was performed using a random number generator.

General anesthesia was induced with a combination of midazolam and propofol intravenously, and the animal was endotracheally intubated. Animals were further instrumented with a jugular central venous line (CVL) and sheath for Swan Ganz catheter, femoral arterial line, nasogastric tube, urinary catheter, and bilateral pleural drains. Surgical tracheostomy was performed in all animals and lung-protective ventilation was applied as according to the EXPRESS trial [50]. After completion of instrumentation, the animal rested for 1 h, then ARDS was induced as follows: (a) in Ph1: sequential administration of oleic acid in subsequent 0.03 mL/kg doses (O1008; Sigma-Aldrich, Castle Hill, Australia) intravenously (IV) through the distal end of the CVL until a PaO_2_/FiO_2_ ratio (PF) of <150 mmHg was reached, and (b) in Ph2: aforementioned oleic acid IV until PF ratio <150 followed by 0.5 µg/kg of lipopolysaccharide (LPS: E. coli O55:B5, Sigma-Aldrich, Castle Hill, Australia), dissolved in 50 mL of normal saline and infused over 1 h. A schematic of the experimental timeline is provided in Figure S5 (Supplementary Materials).

Intra-experimental monitoring, management, and data collection have been reported in detail before [27].

### 4.2. Sample Collection and Processing

Arterial blood samples for protein analysis were collected in EDTA blood tubes at baseline, 2, 6, 12, and 24 h. Samples were centrifuged twice at 3000× *g* for 15 min at 4 °C, and then plasma was aliquoted and stored at −80 °C until batch protein extraction.

Blood samples for full blood count (Mindray Hematology analyzer BC 5000, Nanshan, China) and biochemistry (IDEXX Laboratories Brisbane, Australia) were collected at baseline and every 12 h following T0. In-house ELISAs [51] were used to quantify serum concentrations of inflammatory cytokines (e.g., interleukin (IL) -6, -8, -10).

For histological assessment, lung tissue was collected in 10% neutral buffered formalin for 24 h and then embedded in paraffin. After sectioning to 5 µm thickness, samples were stained with hematoxylin and eosin. All slides were assessed by a blinded, independent veterinary pathologist using the LIS, as recommended by the ATS for experimental ARDS in animal models [48].

### 4.3. Protein Digestion Using Filter-Aided Sample Preparation (FASP) Method

Total protein concentrations of plasma samples were quantified by the Pierce^TM^ BCA protein assay kit (Thermo Fisher Scientific Inc., Waltham, MA, USA) as per the manufacturer’s protocol. In brief, 12.5 µL of sample was added to 12.5 µL of 1× phosphate-buffered saline (PBS), mixed with 200 µL of working BCA reagent in a 96-well microplate, and incubated for 30 min at 37 °C. The absorbance was read at 562 nm on a plate reader and the concentration was calculated against a standard curve ranging from 20 to 2000 µg/mL. Then, 100 µg of total protein was processed for each sample using the FASP method to generate tryptic peptides [52]. Plasma samples were loaded onto spin filter columns (Vivacon^®^ 500–10 kDa MWCO column; Sartorius AG, Gottingen, Germany) and spun at 14,000× *g* at 4 °C for 15 min. Detergent removal by buffer exchange was performed in four successive washes with 8 M urea in 0.1 M Tris-HCl pH 8.5 (200 µL/wash) with a 15 min spin at 14,000× *g* at 4 °C. Afterwards, proteins were reduced using 100 uL of 5 mM DDT in 100 uL of wash buffer and incubated at 56 °C for 30 min, followed by alkylation with 25 mM iodoacetamide (IAA) for 30 min in the dark. Excess IAA was quenched by the addition of 1 µL of 1 M DTT and columns were spun at 14,000× *g* for 15 min. Protein digestion was achieved by adding proteomics-grade trypsin (Trypsin Gold, Promega, Madison, WI, USA) at a 1:25 enzyme to protein ratio in 100 µL of 50 mM ammonium bicarbonate and by incubating at 37 °C overnight. Peptides were recovered by centrifugation and an additional wash with 50 µL of 0.5 M NaCl (14,000× *g* for 5 min each).

### 4.4. Peptide Clean-Up

Digested peptides were then subjected to Zip Tip clean-up (Zip Tip C18, Merck, Darmstadt, Germany). In short, the Zip Tips were activated by passing through 10µL of 80% acetonitrile (ACN) and 0.1% trifluoroacetic acid (TFA) and equilibrated with 10 µL of 1% ACN, 0.1% TFA. The acidified samples were loaded to the Zip Tip, followed by three subsequent washes with 10 µL of 1% ACN, 0.1%TFA. Samples were eluted with 10 µL of 80% ACN, 0.1% TFA. The eluted peptides were dried using a Speed Vac vacuum concentrator and reconstituted in 0.1% TFA to a final concentration of 5 µg peptide/100 µL 0.1% TFA. Samples were then frozen at −80 °C until mass spectrometric analysis.

### 4.5. Mass Spectrometry

Liquid chromatography–mass spectrometry (LC–MS/MS) analysis was performed using a Prominence nanoLC system (Shimadzu) and 5600 Triple-TOF mass spectrometer with a Nanospray III interface (AB Sciex, Framingham MA, USA). First, 0.5–2 μg peptides were desalted on an Agilent C18 trap (300 Å pore size, 5 μm particle size, 0.3 mm i.d. Å~5 mm) at a flow rate of 30 μL/min for 3 min, and then separated on a Vydac EVEREST reversed-phase C18 high-performance liquid chromatography (HPLC) column (300 Å pore size, 5 μm particle size, 150 mm × 150 μm i.d. Å~ 150 mm) at a flow rate of 1 μL/min [53]. Separation of peptides was done by using a binary solvent system: gradient of 5–35% buffer B over 45 min (buffer A: 1% acetonitrile + 0.1% formic acid; buffer B: 80% acetonitrile with 0.1% formic acid). Settings of gas and voltage were adapted as necessary.

An MS-TOF scan at *m/z* of 350–1800 was performed for 0.5 s, followed by data-dependent acquisition (DDA) of MS/MS with automated CE selection of the top 20 peptides at *m/z* of 400–1250 for 0.5 s per spectrum. SWATH-MS was done with LC conditions as before, but with an MS-TOF scan at an *m/z* of 350–1800 for 0.05 s, followed by high-sensitivity information-independent acquisition with 26 *m/z* isolation windows, with 1 *m/z* window overlap each for 0.1 s across an * m/z* range of 400–1250.

### 4.6. Data Processing and Quality Control

A spectral library was generated in ProteinPilot (v5.0, AB Sciex) using the DDA files, and peptides were identified, searching the UniProt database (downloaded from http://www.uniprot.org as of 8 October 2021, 162`326 sequences; accessed on 8 October 2021) with standard settings: sample type: identification; alkylation: iodoacetamide; digestion: trypsin; instrument: TripleTOF 5600; special factors: none; ID focus: biological modifications; search effort: thorough ID; detected protein threshold: >0.05 (10.0%). Determined by a confidence of >99% and a global FDR rate of <1%, the spectral library contained 5907 peptides corresponding to 226 proteins.

SWATH data were analyzed in PeakView (v2.1, AB Sciex) and transition ion, peptide, and protein peak areas were extracted and exported for further analysis. Data were reformatted for statistical analysis as previously described [54].

Peak protein intensity areas were further processed with MSstats: after log2 normalization, quality control was performed. Features with one or two measurements across runs were removed, and log2 intensities under the cutoff of 3.2678 and NA values were considered as censored missing values. There were 198 proteins consisting of 1–12 peptides per protein and 1–6 features per peptide available for further analysis.

### 4.7. Statistical Analysis

Proteins that were expressed in at least 4/5 Ph1 and 3/4 Ph2 samples at each assessed time point were analyzed for differential abundance among Ph2 and Ph1 and compared with a Wilcoxon test.

Clusters among subphenotypes and time points were visualized using principal component analysis (PCA). To detect a protein signature for differentiating between Ph1 and Ph2, a partial least squares discriminant analysis (PLS-DA) was performed, a supervised clustering method for predictive and descriptive modeling as well as for discriminative variable selection.

For proteins that were detected in at least three time points in each animal, linear-mixed-effects models (LMM) [55,56] were constructed to assess levels of proteins over time among the two groups. Distribution of data was assessed with QQ and residual plots. The assessed protein over time was included in the model as the dependent variable. As the structure of random effects, the individual animal (1 | ID) was used throughout. Fixed effects were reported as estimates with 95% confidence intervals (CI) for phenotypes, time (in hours), and interaction phenotype:time.

The STRING database (STRING Consortium 2018; https://www.string-db.org/) was consulted to analyze protein–protein interaction (PPI) networks. This database displays known and predicted protein interactions based on direct and indirect (functional) associations.

All hypothesis testing was two-tailed. All statistical analyses were performed with R Version 4.0.5 (R Foundation for Statistical Computing, Vienna, Austria), using the packages “MSstats”, “mixOmics”, “ggvenn”, “lmerTest”, and “stargazer”.

## 5. Conclusions

Among the two phenotypes of ovine ARDS, patterns of proteins known to be involved in biological processes associated with ARDS emerge over time. Additionally, it is possible to derive a characteristic protein signature to differentiate between the two phenotypes.

## Figures and Tables

**Table 1:** **A**–**C:** component loadings for components 1 to 3.

(A) Component 1	Ph1	Ph2	GroupContrib	Importance
hypothetical protein JEQ12_008126	−0.50842	0.635523	Ph2	−0.49715
hypothetical protein JEQ12_002713	−0.46116	0.576452	Ph2	−0.4048
immunoglobulin J chain	−0.43948	0.549347	Ph2	−0.36243
hypothetical protein JEQ12_001510	−0.40883	0.511038	Ph2	−0.30254
lumican	−0.46124	0.569764	Ph2	−0.26074
alpha-1-macroglobulin-like isoform X1	−0.4179	0.459686	Ph2	−0.22226
hypothetical protein JEQ12_014972	−0.36751	0.459382	Ph2	−0.22178
clusterin	−0.36404	0.455049	Ph2	−0.21501
hypothetical protein JEQ12_010483	−0.35937	0.449214	Ph2	−0.20589
inter-alpha-trypsin inhibitor heavy chain H2 isoform X2	−0.34194	0.427429	Ph2	−0.17183
hypothetical protein JEQ12_008129, partial	−0.35521	0.448681	Ph2	−0.16998
hypothetical protein JEQ12_008015	−0.33536	0.419202	Ph2	−0.15897
heparin cofactor 2	−0.32167	0.386004	Ph2	−0.10707
adiponectin isoform X1	−0.36314	0.453928	Ph2	−0.07133
hemoglobin subunit beta	0.279596	−0.34949	Ph1	0.049993
hypothetical protein JEQ12_003887	−0.27908	0.348852	Ph2	−0.04899
sex hormone-binding globulin isoform X3	−0.34802	0.386691	Ph2	−0.04769
fibronectin isoform X8	−0.2735	0.34187	Ph2	−0.03807
complement component C8 gamma chain	−0.39878	0.451948	Ph2	−0.03353
short palate, lung and nasal epithelium carcinoma-associated protein 2B-like	−0.2696	0.37445	Ph2	−0.03047
**(B) Component 2**	**Ph1**	**Ph2**	**GroupContrib**	**Importance**
fibrinogen gamma chain isoform X1	0.244913	−0.30614	Ph1	0.995488
hemopexin	0.093814	−0.11727	Ph1	0.089809
hypothetical protein JEQ12_017492	0.083199	−0.104	Ph1	0.030618
**(C) Component 3**	**Ph1**	**Ph2**	**GroupContrib**	**Importance**
serum paraoxonase/arylesterase 1 isoform X1	−0.04943	0.061784	Ph2	0.562009
retinol-binding protein 4	0.056336	−0.07116	Ph1	0.49437
serpin A3–8	−0.21267	0.265832	Ph2	−0.39709
alpha-2-macroglobulin isoform X3	−0.22798	0.28498	Ph2	0.350947
thyroxine-binding globulin precursor	0.11401	−0.10801	Ph1	0.255706
hypothetical protein JEQ12_010483	−0.35937	0.449214	Ph2	−0.18727
carboxypeptidase N subunit 2	0.119992	−0.10736	Ph1	0.141224
PHD finger protein 21A isoform X13	0.07898	−0.09478	Ph1	0.133177
immunoglobulin lambda variable 1–40 isoform X18	0.047427	−0.06324	Ph1	0.130715
hypothetical protein JEQ12_012143	0.229434	−0.28679	Ph1	0.060661

Abbrev: Ph1/2: phenotypes 1 and 2; GroupContrib: group contribution.

**Table 2:** **A**–**C:** Estimates of linear mixed-effect models.

(A) *p* < 0.1 among Ph1 and Ph2	Ph1/Ph2	Time	Interaction	Constant
immunoglobulin J chain	0.9 (−0.09, 1.88), *p* < 0.1	0.002 (−0.13, 0.14)	−0.02 (−0.22, 0.18)	9.72 (9.06, 10.37), *p* < 0.01
heparin cofactor 2	0.9 (−0.0004, 1.80), *p* < 0.1	−0.09 (−0.27, 0.09)	−0.12 (−0.39, 0.15)	10.17 (9.57, 10.77), *p* < 0.01
immunoglobulin lambda-1 light chain isoform X47	−1.35 (−2.85, 0.16), *p* < 0.1	−0.43 (−1.38, 0.53)	0.41 (−1.02, 1.84)	5.37 (4.37, 6.37), *p* < 0.01
angiotensinogen	0.88 (−0.14, 1.89), *p* < 0.1	−0.12 (−0.30, 0.06)	−0.16 (−0.43, 0.11)	10.16 (9.48, 10.84), *p* < 0.01
hypothetical protein JEQ12_001510	0.84 (0.18, 1.50), *p* < 0.05	0.06 (−0.06, 0.17)	−0.11 (−0.28, 0.06)	11.02 (10.57, 11.46), *p* < 0.01
**(B) *p* < 0.1 over time**	**Ph1/Ph2**	**Time**	**Interaction**	**Constant**
apolipoprotein C-III	−0.14 (−1.73, 1.46)	−0.48 (−0.79, −0.16), *p* < 0.01	0.17 (−0.31, 0.64)	9.51 (8.45, 10.58), *p* < 0.01
ceruloplasmin isoform X2	0.09 (−0.78, 0.96)	0.1 (−0.02, 0.23), *p* < 0.1	0.02 (−0.17, 0.20)	12.34 (11.76, 12.91), *p* < 0.01
complement C4-like isoform X1	0.01 (−1.06, 1.09)	−0.25 (−0.43, −0.06), *p* < 0.05	−0.03 (−0.31, 0.26)	11.02 (10.31, 11.74), *p* < 0.01
inter-alpha-trypsin inhibitor heavy chain H2 isoform X2	0.61 (−0.14, 1.36)	−0.22 (−0.33, −0.12), *p* < 0.01	−0.02 (−0.18, 0.14)	11.57 (11.07, 12.07), *p* < 0.01
prothrombin precursor	0.34 (−1.10, 1.79)	−0.27 (−0.48, −0.05), *p* < 0.05	0.08 (−0.24, 0.40)	10.98 (10.02, 11.95), *p* < 0.01
serpin A3–7 isoform X2	0.16 (−0.63, 0.95)	0.19 (0.05, 0.34), *p* < 0.01	−0.1 (−0.32, 0.12)	10.36 (9.83, 10.88), *p* < 0.01
serpin A3–8	0..35 (−0.84, 1.53)	0.47 (0.28, 0.65), *p* < 0.01	0.07 (−0.20, 0.35)	8.86 (8.07, 9.65), *p* < 0.01
serpin A3–6-like	−1.55 (−4.04, 0.93)	−0.42 (−0.89, 0.05), *p* < 0.1	0.28 (−0.40, 0.95)	9.63 (7.92, 11.35), *p* < 0.01
serum amyloid A protein	0.29 (−1.64, 2.21)	1.42 (1.06, 1.79), *p* < 0.01	−0.03 (−0.55, 0.49)	4.09 (2.73, 5.46), *p* < 0.01
glutathione peroxidase 3	−0.39 (−1.82, 1.04)	0.27 (−0.01, 0.55), *p* < 0.1	0.31 (−0.07, 0.70)	5.69 (4.64, 6.74), *p* < 0.01
synaptotagmin-like protein 4 isoform X3	−0.21 (−1.10, 0.69)	0.18 (0.01, 0.34), *p* < 0.05	−0.01 (−0.26, 0.24)	16.43 (15.84, 17.03), *p* < 0.01
serum paraoxonase/arylesterase 1 isoform X1	0.35 (−0.38, 1.08)	−0.11 (−0.22, 0.01), *p* < 0.1	−0.1 (−0.27, 0.08)	11.6 (11.11, 12.08), *p* < 0.01
transthyretin precursor	−1.35 (−4.77, 2.06)	−0.48 (−0.97, 0.01), *p* < 0.1	0.1 (−0.67, 0.87)	11.47 (9.21, 13.73), *p* < 0.01
lumican	0.2 (−1.22, 1.62)	0.35 (0.07, 0.62), *p* < 0.05	0.47 (0.06, 0.87), *p* < 0.05	6.57 (5.08, 7.02), *p* < 0.01
zinc finger protein 264-like isoform X1	0.28 (−0.58, 1.14)	0.17 (−0.01, 0.36), *p* < 0.1	−0.2 (−0.46, 0.06)	12.15 (11.54, 12.75), *p* < 0.01
Hemopexin	−0.8 (−2.22, 0.61)	−0.38 (−0.63, −0.14), *p* < 0.01	0.19 (−0.18, 0.55)	12.93 (11.98, 13.87), *p* < 0.01
complement C3	0.08 (−0.66, 0.82)	−0.12 (−0.25, 0.01), *p* < 0.1	−0.001 (−0.20, 0.20)	9.53 (9.03, 10.02), *p* < 0.01
hemoglobin subunit beta	−1.74 (−4.02, 0.53)	0.44 (0.02, 0.85), *p* < 0.05	0.17 (−0.45, 0.80)	13.25 (11.74, 14.77), *p* < 0.01
apolipoprotein A-II	0.58 (−0.45, 1.61)	−0.18 (−0.38, 0.03), *p* < 0.1	−0.08 (−0.39, 0.23)	12.09 (11.40, 12.78), *p* < 0.01
PHD finger protein 21A isoform X13	−0.05 (−1.18, 1.08)	0.22 (0.001, 0.44), *p* < 0.05	−0.04 (−0.37, 0.29)	14.89 (14.13, 15.64), *p* < 0.01
hypothetical protein JEQ12_002713	0.54 (−0.15, 1.22)	−0.11 (−0.24, 0.01), *p* < 0.1	0.03 (−0.16, 0.22)	12.24 (11.78, 12.69), *p* < 0.01
hypothetical protein JEQ12_008125	−0.42 (−1.05, 0.22)	−0.15 (−0.26, −0.04), *p* < 0.01	0.14 (−0.03, 0.31)	17.7 (17.28, 18.12), *p* < 0.01
hypothetical protein JEQ12_008126	0.4 (−0.19, 1.00)	−0.12 (−0.21, −0.02), *p* < 0.05	0.08 (−0.06, 0.22)	15.48 (15.09, 15.88), *p* < 0.01
hypothetical protein JEQ12_008387	0.34 (−0.33, 1.01)	0.53 (0.39, 0.66), *p* < 0.01	−0.05 (−0.25, 0.15)	10.18 (9.73, 10.62), *p* < 0.01
hypothetical protein JEQ12_005133	−0.17 (−1.93, 1.58)	1.72 (1.39, 2.04), *p* < 0.01	0.02 (−0.49, 0.53)	3.73 (2.63, 4.83), *p* < 0.01
hypothetical protein JEQ12_003887	0.59 (−0.72, 1.91)	−0.22 (−0.46, 0.02), *p* < 0.1	0.03 (−0.33, 0.38)	13.07 (12.20, 13.95), *p* < 0.01
hypothetical protein JEQ12_012143	−0.51 (−2.55, 1.53)	0.58 (0.17, 0.99), *p* < 0.01	−0.13 (−0.74, 0.49)	13.01 (11.65, 14.37), *p* < 0.01
**(C) *p* < 0.1 for group:time interaction**	**Ph1/Ph2**	**Time**	**Interaction**	**Constant**
lumican	0.2 (−1.22, 1.62)	0.35 (0.07, 0.62), *p* < 0.05	0.47 (0.06, 0.87), *p* < 0.05	6.57 (5.08, 7.02), *p* < 0.01
carbonic anhydrase 2	−1.36 (−3.16, 0.43)	0.17 (−0.13, 0.47)	0.39 (−0.06, 0.83), *p* < 0.1	9.62 (8.41, 10.83), *p* < 0.01

Data are expressed as estimates with 95% confidence interval in brackets.

**Table 3:** Protein identification with protein BLAST.

Input in BLAST	Identified Protein	Accession Number	Perc. Identity	Query Cover
hypothetical protein JEQ12_001510	kininogen-1 isoform X2	XP_004003107.2	99.77%	100%
hypothetical protein JEQ12_002713	primary amine oxidase, liver isozyme	XP_027830273.2	99.86%	100%
hypothetical protein JEQ12_008126	immunoglobulin mu chain	AAA51379.1	99.79%	73%
hypothetical protein JEQ12_008387	inter-alpha-trypsin inhibitor heavy chain H4 isoform X2	XP_004018440.3	99.56%	100%
hypothetical protein JEQ12_005133	haptoglobin isoform X2	XP_004015160.1	99.75%	100%
hypothetical protein JEQ12_003887	antithrombin-III precursor	NP_001009393.1	99.57%	100%
hypothetical protein JEQ12_012143	hemoglobin subunit alpha	EGW10374.1	100%	97%
hypothetical protein JEQ12_008125	Ig gamma 1 chain	CAA49451.1	99.70%	81%

## Data Availability

The data presented in this study are available on request from the corresponding author. The data are not publicly available due to being part of a PhD project.

## Supplementary Materials

The following supporting information can be downloaded at: https://www.mdpi.com/article/10.3390/metabo12070655/s1, Figure S1: Cytokine levels in plasma among phenotypes at different time points during observation time, Figure S2: Histopathological assessment of the lungs at study end, Figure S3: Correlation plot of all samples, Figure S4: PLS-DA: features per component and initial error rate, Figure S5 Study design and time line, Table S1: Baseline characteristics, Table S2: Clinical and laboratory parameters at T12 and T24, Table S3: Differentially expressed proteins among Ph1 and Ph2 at every time point, Table S4: Biological processes and associated proteins in pathway analysis.

## Abbreviations

ACN	acetonitrile
AGT	angiotensinogen
APOA2	apolipoprotein A-II
APOC3	apolipoprotein C-III
ARDS	acute respiratory distress syndrome
ATS	American Thoracic Society
AUC	area under the curve
BCA	bicinchoninic acid
BLAST	Basic Local Alignment Search Tool
CA2	carbonic anhydrase 2
COVID-19	coronavirus disease 2019
CP	ceruloplasmin precursor
CVL	central venous line
DDA	data-dependent acquisition
DTT	dithiothreitol
ENSOARP00000000771	uncharacterized protein
ENSOARP00000002890	complement C4-like isoform X1
ENSOARP00000011736	hemoglobin subunit alpha
ENSOARP00000016410	uncharacterized protein, belongs to serpin family
F2	thrombin
FDR	false discovery rate
FiO_2_	fraction of inspired oxygen
GPX3	glutathione peroxidase 3
HBB	hemoglobin subunit beta
HPLC	high-performance liquid chromatography
HPX	hemopexin
IAA	iodoacetamide
ITIH2	inter-alpha-trypsin inhibitor heavy chain H2
KNG1	kininogen-1 isoform X2
LC–MS/MS	liquid chromatography–mass spectrometry
LIS	Lung Injury Score
LMM	linear-mixed effects models
LOC101113086	primary amine oxidase, lung isozyme
LPS	lipopolysaccharide
LUM	lumican
MERF	Medical Engineering Research Facility
MS TOF	mass spectrometry time-of-flight
MWCO	molecular weight cutoff
NHLBI	National Health Lung and Blood Institute
OA	oleic acid
P1	human hypoinflammatory subphenotype
P2	human hyperinflammatory subphenotype
PaO_2_	partial pressure of oxygen in arterial blood
PC	principal component
PCA	principal component analysis
PF ratio	PaO_2_/FiO_2_ ratio
Ph1	phenotype 1
Ph2	phenotype 2
PHF21A	pHD finger protein 21A
PPI	protein–protein interaction
PLS-DA	partial least squares-discriminant analysis
SAA1	serum amyloid A protein
SERPINC1	antithrombin-III precursor
STRING	search tool for the retrieval of interacting genes/proteins
SWATH	sequential window acquisition of all theoretical mass spectra
TFA	trifluoroacetic acid
TOF	time of flight
TTR	transthyretin
QUT	Queensland University of Technology
