# Supplementary material for: Differential Protein Expression among Two Different Ovine ARDS Phenotypes—A Preclinical Randomized Study
Source: Metabolites. 2022 Jul 15;12(7):655. doi: 10.3390/metabo12070655 (PMC9319228; doi:10.3390/metabo12070655)
Supplement: Supplementary file 1 [file metabolites-12-00655-s001.zip › metabolites-1785679-supplementary.pdf]

ONLINE SUPPLEMENT

Supplemental Results

1) **Table S1 Baseline characteristics**
2) **Table S2 Clinical and laboratory parameters at T12 and T24**
3) **Figure S1 Cytokine levels in plasma among phenotypes at different time points during observation time**
4) **Figure S2 Histopathological assessment of the lungs at study end**
5) **Figure S3 Correlation plot of all samples**
6) **Table S3 Differentially expressed proteins among Ph1 and Ph2 at every time point**
7) **Figure S4 PLS-DA: features per component and initial error rate**
8) **Table S4 Biological processes and associated proteins in pathway analysis**

Supplemental Methods

1) **Figure S5 Study design and time line**
2) **Adherence to main features of experimental ALI according to the ATS definition**

Supplemental Results

Table S1 Baseline characteristics

.	all	Ph1, n=5	Ph2, n=4	p
Weight (kg)	48 (44-51)	48 (45-52)	47 (43-52)	0.9
Body surface area (per m^2)	1.3 (1.2-1.3)	1.3 (1.2-1.3)	1.2 (1.2-1.3)	0.9
Temperature (°C)	38.7 (37.9-38.9)	38.1 (37.7-38.7)	38.9 (38.0-39.4)	0.2
Hemodynamic parameters				
Mean arterial blood pressure (mmHg)	122 (90-129)	122 (88-127)	121 (83-130)	0.7
Heart rate (bpm)	72 (63-103)	90 (63-105)	72 (55-95)	0.6
Cardiac index	4.4 (3.3-5.3)	4.5 (3.2-6.6)	4.3 (3.4-5.4)	0.7
Mechanical ventilation				
Minute ventilation (L/min)	6.4 (5.5-6.9)	6.1 (5.3-6.9)	6.6 (5.7-6.9)	0.6
Compliance (mL/cmH_2O)	33.4 (26.5-42.3)	33.4 (26.7-53.0)	35.0 (23.8-40.0)	0.7
PEEP (cmH20)	5 (5-5)	5 (5-5)	5 (5-5)	1.0
Plateau pressure (cm H_2O)	15 (13-20)	15 (13-22)	15 (13-22)	1.0
Extravascular lung water index	27 (24-32)	27 (18-37)	28 (25-29)	1.0
Blood gases				
PaO2/FiO2 ratio	494 (412-528)	492 (284-513)	518 (499-533)	0.3
PaCO2 (mmHg)	41 (40-44)	43 (41-47)	41 (39-41)	0.1
Oxyhemoglobin	98 (98-99)	98 (96-99)	98 (98-99)	0.7
Lactate	0.8 (0.4-1.0)	0.8 (0.4-1.1)	0.7 (0.4-0.9)	0.7
Base excess (mmol/L)	2.0 (-0.2-3.9)	2.0 (0.4-4.7)	1.2 (-0.3-3.5)	0.4
Bicarbonate (mmol/L)	27 (24-28)	27 (25-29)	25 (23-27)	0.3
Full blood count				
Hemoglobin (g/L)	113 (105-121)	118 (104-122)	109 (105-119)	0.7
Platelets (10^9/L)	364 (250-413)	278 (204-413)	368 (278-426)	0.6
Neutrophil count (10^9/L)	1.8 (1.6-2.2)	2.1 (1.6-2.8)	1.7 (1.5-1.9)	0.3
Lymphocyte count (10^9/L)	2.9 (2.6-3.6)	2.9 (2.6-4.0)	2.9 (2.5-3.2)	0.7
Biochemistry				
Sodium (mmol/L)	143 (141-145)	143 (141-145)	142 (140-144)	0.4
Potassium (mmol/L)	4.7 (4.3-4.8)	4.3 (4.3-4.7)	4.8 (4.6-5.7)	0.1
Creatinine (mmol/L)	0.06 (0.06-0.08)	0.08 (0.06-0.08)	0.06 (0.05-0.08)	0.3
Bilirubin (umol/L)	4 (3-4)	3 (3-4)	4 (3-4)	0.1
ASAT (IU/L)	93 (85-108)	90 (77-99)	105 (93-134)	0.1
ALP (IU/L)	154 (121-294)	148 (74-213)	247 (140-343)	0.3
CK (IU/L)	192 (119-240)	128 (100-228)	214 (171-250)	0.2
Albumin (g/L)	34.0 (32.5-36.5)	34.0 (32.5-37.0)	34.0 (32.3-36.5)	0.9
Cytokines				
IL-6 plasma (pg/ml)	313 (0.4)	313 (0.4)	313 (0.5)	0.9
IL-8 plasma (pg/ml)	875 (625)	1248 (573)	409 (286)	0.03
IL-10 plasma (pg/ml)	992 (497)	985 (459)	999 (614)	1.0

Parameters displayed as median and IQR, cytokine levels as mean and standard deviation.

Abbreviations: Ph1 and Ph2: phenotype 1 and 2; PEEP: positive end-expiratory pressure; PaCO2 (mmHg): arterial carbondioxide partial pressure; ASAT: aspartate transaminase; ALP: alkaline phosphatase; CK: creatin kinase, IL: interleukin.

Table S2 Clinical and laboratory parameters at T12 and T24

	at T12			at T24		
	**all, n=9**	**Ph1, n=5**	**Ph2, n=4**	**all, n=9**	**Ph1, n=5**	**Ph2, n=4**
Temperature (°C)	38.9 (37.8-39.4)	39.8 (37.3-39.2)	39.3 (38.1-39.9)	38.7 (38.2-39.4)	38.6 (38.2-39.1)	39.2 (38.1-39.9)
Hemodynamic parameters						
Mean arterial blood pressure (mmHg)	71 (61-74)	71 (54-74)	71 (66-80)	76 (63-78)	64 (58-78)	77 (72-81)
Use of noradrenaline (mcg/kg/min)	0.17 (0.10-0.27)	0.17 (0.03-0.27)	0.19 (0.15-0.33)	0.25 (0.17-0.34)	0.25 (0.21-0.29)	0.28 (0.15-0.39)
Heart rate (bpm)	107 (61-74)	105 (96-130)	124 (96-142)	122 (101-128)	116 (101-126)	122 (99-133)
Cardiac index	4.9 (3.7-6.2)	3.9 (3.5-6.2)	5.6 (5.0-6.4)	6.5 (5.8-7.4)	6.2 (5.4-6.9)	7.1 (6.0-9.0)
Mechanical ventilation						
Minute ventilation (L/min)	10.8 (9.1-11.9)	10.7 (8.4-11.0)	11.9 (9.2-14.9)	14.7 (10.8-15.8)	13.8 (10.8-15.2)	15.4 (11.4-17.0)
Compliance (mL/cmH $_2$ O)	17.4 (14.7-21.0)	15.6 (13.3-23.7)	17.7 (15.7-19.5)	19.0 (15.5-22.4)	18.5 (13.5-20.4)	21.0 (16.6-25.0)
Plateau pressure (cm H_2O)	22 (21-26)	22 (19-27)	22.5 (21.8-27.4)	26 (21-28)	26 (21-28)	24 (20-28)
Extravascular lung water index	49 (42-55)	55 (39-55)	49 (39-54)	50 (43-55)	50 (32-55)	48 (42-54)
Blood gases						
PaO2/FiO2 ratio	272 (209-324)	220 (174-290)	295 (273-329)	268 (216-309)	258 (136-278)	299 (265-339)
PaCO2 (mmHg)	47 (42-58)	47 (42-60)	48 (39-56)	52 (41-59)	52 (41-57)	53 (40-63)
Lactate	2.4 (2.0-4.3)	2.3 (2.0-3.3)	3.6 (1.7-6.5)	2.6 (1.6-4.3)	2.3 (1.6-2.6)	3.8 (1.3-8.7)
Base excess (mmol/L)	-0.8 (-3.4-0.1)	0 (-1.9-0.5)	-2.4 (-4.3—0.1)	-3.2 (-4.3—1.8)	-3.2 (-3.9-0.4)	-3.7 (-6.6—2.4)
Full blood count						
Hemoglobin (g/L)	113 (97-123)	113 (96-118)	113 (96-126)	108 (98-115)	108 (103-117)	103 (92-113)
Platelets (10^9/L)	237 (142-267)	250 (189-267)	176 (82-271)	188 (91-223)	217 (123-234)	140 (51-217)
Neutrophil count (10^9/L)	1.2 (0.8-1.5)	0.8 (0.7-1.2)	1.5 (1.2-2.0)	0.7 (0.3-1.3)	0.4 (0.2-0.7)	1.3 (0.6-1.5)
Lymphocyte count (10^9/L)	0.9 (0.8-1.3)	1.0 (0.6-1.4)	0.9 (0.9-1.2)	1.4 (0.9-1.8)	1.2 (0.9-1.8)	1.4 (0.9-1.9)
Biochemistry						
Sodium (mmol/L)	144 (143-147)	144 (142-148)	145 (143-147)	146 (144-150)	146 (144-151)	147 (144-151)
Potassium (mmol/L)	5.3 (4.8-6.5)	4.9 (4.4-6.3)	5.8 (5.0-6.6)	5.3 (4.9-6.3)	5.3 (4.8-6.2)	5.4 (4.9-6.8)
Creatinine (mmol/L)	0.12 (0.08-0.15)	0.12 (0.08-0.15)	0.14 (0.09-0.16)	0.14 (0.11-0.25)	0.14 (0.12-0.26)	0.15 (0.11-0.24)
Bilirubin (umol/L)	6 (2-13)	4 (0.5-15)	8 (4-14)	8 (6-19)	6 (4-20)	9 (7-19)
ASAT (IU/L)	196 (160-242)	180 (139-233)	215 (192-314)	353 (295-501)	353 (261-477)	383 (317-544)
ALP (IU/L)	107 (62-184)	80 (35-122)	184 (100-249)	89 (52-195)	75 (31-123)	195 (96-224)
CK (IU/L)	8119 (4597-12030)	6709 (2150-9514)	11157 (8557-14990)	21462 (15409-25673)	21462 (12105-31112)	21610 (18656-24386)
Albumin (g/L)	21.0 (19.5-22.0)	22.0 (21.0-22.5)	19.5 (16.5-21.0)	20.0 (17.0-21.5)	21.0 (18.5-22.0)	18.5 (14.8-20.8)
Cytokines						
IL-6 plasma (pg/ml)	217133 (183445)	1105456 (81890)	350480 (195261)	198673 (151525)	127902 (59632)	287137 (194173)
IL-8 plasma (pg/ml)	815 (658)	918 (865)	685 (341)	1524 (1430)	1916 (1562)	1034 (1275)
IL-10 plasma (pg/ml)	2458 (925)	2482 (865)	2427 (1106)	2789 (892)	2922 (940)	2625 (938)

Parameters displayed as median and IQR, cytokine levels as mean and standard deviation.

Abbreviations: Ph1 and Ph2: phenotype 1 and 2; PaCO2 (mmHg): arterial carbondioxide partial pressure; ASAT: aspartate transaminase; ALP: alkaline phosphatase; CK: creatine kinase, IL: interleukin; IFNy: interferon gamma.

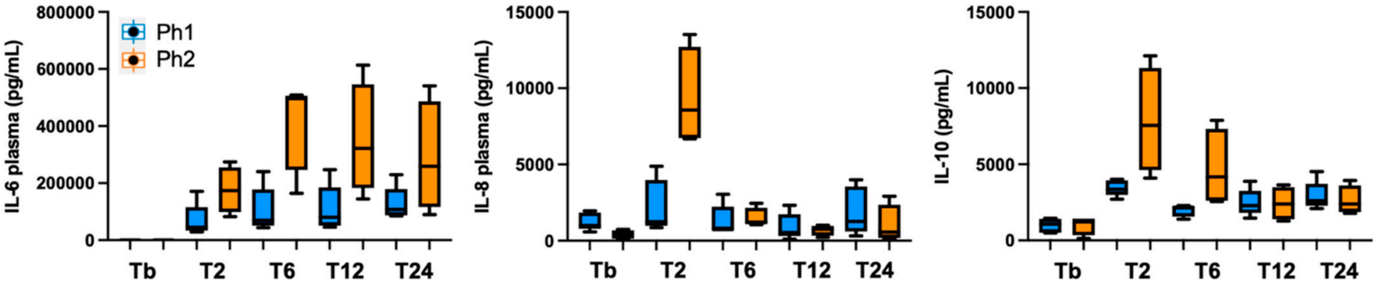

Figure S1 Cytokine levels in plasma among phenotypes at different time points during observation time. Abbreviations: Ph1 and Ph2: phenotype 1 and 2; IL: interleukin; Tb: baseline; T: time point.

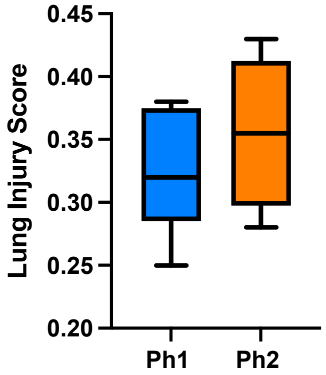

Figure S2 Histopathological assessment of the lungs at study end. Abbreviations: Ph1 and Ph2: phenotype 1 and 2

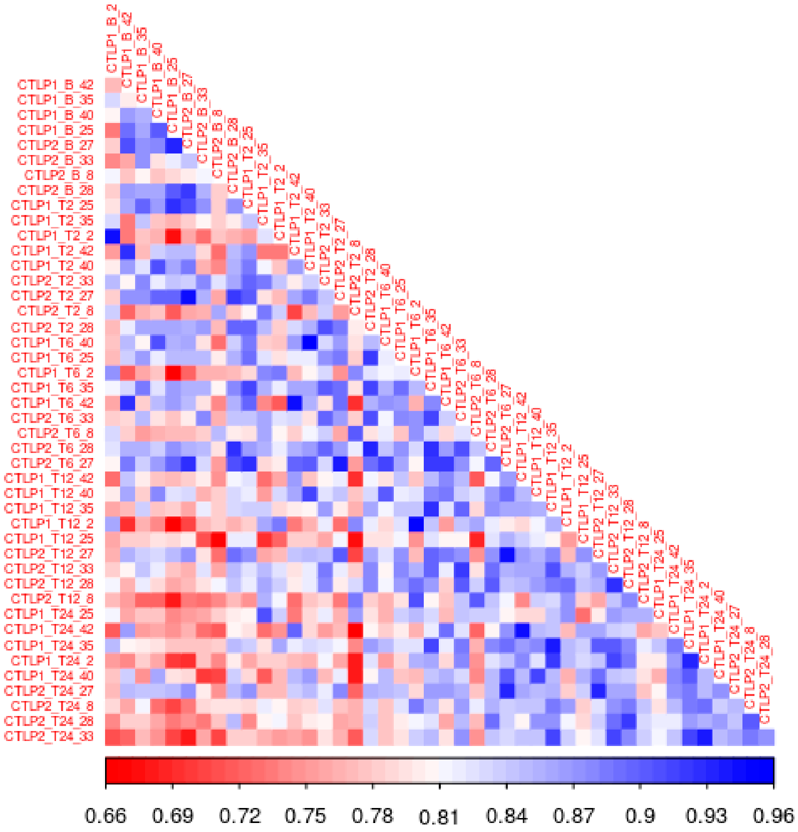

Figure S3 Correlation plot of all samples**.** Abbreviations: P1 and P2 = phenotype 1 and 2; CTL = control; T = time point

Table S3 Differentially expressed proteins among Ph1 and Ph2 at every time point

Baseline	protein	p (wilc)
	apolipoprotein A-II [Ovis aries]	0.015873016
	CD5 antigen-like [Ovis aries]	0.015873016
	complement factor I [Ovis aries]	0.015873016
	hypothetical protein JEQ12_008015 [Ovis aries]	0.015873016
	hypothetical protein JEQ12_008126 [Ovis aries]	0.015873016
	hypothetical protein JEQ12_010483 [Ovis aries]	0.015873016
	hypothetical protein JEQ12_014972 [Ovis aries]	0.015873016
	immunoglobulin J chain [Ovis aries]	0.015873016
	complement component C8 gamma chain [Ovis aries]	0.028571429
	lumican [Ovis aries]	0.028571429
	clusterin [Ovis aries]	0.031746032
	heparin cofactor 2 [Ovis aries]	0.031746032
	hypothetical protein JEQ12_001510 [Ovis aries]	0.031746032
	hypothetical protein JEQ12_002713 [Ovis aries]	0.031746032
	inter-alpha-trypsin inhibitor heavy chain H2 isoform X2 [Ovis aries]	0.031746032
	plasma protease C1 inhibitor [Ovis aries]	0.031746032
**T2**	**Protein**	**p (wilc)**
	synaptotagmin-like protein 4 isoform X3 [Ovis aries]	0.031746032
**T6**	**Protein**	**p (wilc)**
	hypothetical protein JEQ12_002713 [Ovis aries]	0.015873016
	hypothetical protein JEQ12_008129, partial [Ovis aries]	0.028571429
**T12**	**Protein**	**p (wilc)**
	hypothetical protein JEQ12_008126 [Ovis aries]	0.031746032
**T24**	**Protein**	**p (wilc)**
	adiponectin isoform X1 [Ovis aries]	0.015873016
	hypothetical protein JEQ12_008022 [Ovis aries]	0.015873016
	hypothetical protein JEQ12_010483 [Ovis aries]	0.015873016
	immunoglobulin J chain [Ovis aries]	0.015873016
	lumican [Ovis aries]	0.015873016
	plasma protease C1 inhibitor [Ovis aries]	0.015873016
	apolipoprotein E [Ovis aries]	0.028571429
	C4b-binding protein alpha chain isoform X8 [Ovis aries]	0.028571429
	alpha-2-HS-glycoprotein precursor [Ovis aries]	0.031746032
	apolipoprotein A-I isoform X1 [Ovis aries]	0.031746032
	apolipoprotein A-IV [Ovis aries]	0.031746032
	complement factor I [Ovis aries]	0.031746032
	gelsolin isoform X4 [Ovis aries]	0.031746032
	hypothetical protein JEQ12_002713 [Ovis aries]	0.031746032
	hypothetical protein JEQ12_003887 [Ovis aries]	0.031746032
	hypothetical protein JEQ12_008126 [Ovis aries]	0.031746032
	hypothetical protein JEQ12_013893 [Ovis aries]	0.031746032

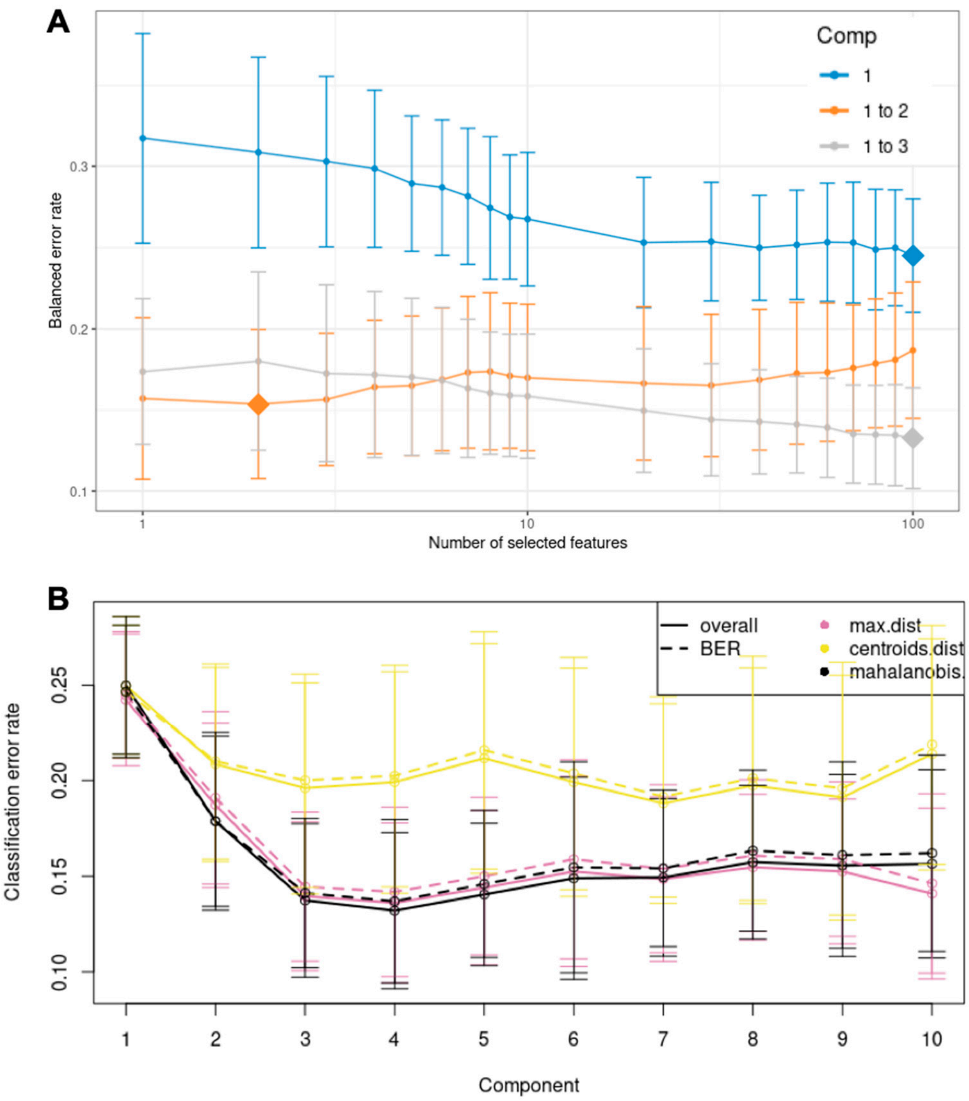

Figure S4 PLS-DA: features per component and initial error rate**.** Abbreviations: Comp: component, BER: balanced error rate; max. dist.: maximal distance; centroids dist.: centroids distance

Table S4 Biological processes and associated proteins in pathway analysis

biological process and involved proteins	strength	FDR
**regulation of cholesterol and lipid metabolism**	2.98	0.0115
APOC3		
APOA2		
**phospholipid efflux**	2.32	0.0301
APOC3		
APOA2		
**Oxygen carrier and binding activity**	2.24	0.027
HBB		
ENSOARP00000011736		
**Complement and coagulation cascades**	1.88	<0.0001
KNG1		
SERPINC1		
F2		
ENSOARP00000002890		
ENSOARP00000000771		
**Negative regulation of endopeptidase activity**	1.35	0.0097
ENSOARP00000016410		
ITIH2		
KNG1		
SERPINC1		
**Inflammatory response**	1.1	0.023
ENSOARP00000002890		
KNG1		
F2		
SAA1		
ENSOARP00000000771		
**Negative regulation of catalytic activity**	1.05	0.0015
ENSOARP00000000771		
ENSOARP00000016410		
SERPINC2		
KNG1		
ITIH2		
APOC3		
APOA2		
**Defense responses**	0.81	0.0314
ENSOARP00000000771		
ENSOARP00000002890		
APOA2		
F2		
KNG1		
SAA1		
IGJ		

Abbreviations: FDR: false discovery rate; APOA2: apolipoprotein A-II; ENSOARP00000000771: uncharacterized protein; ITIH2: inter-alpha-trypsin inhibitor heavy chain H2; HBB: hemoglobin subunit beta; IGJ: immunoglobulin J chain; ENSOARP00000011736: hemoglobin subunit alpha; SAA1: serum amyloid A protein; ENSOARP00000016410: uncharacterized protein, belongs to serpin family; APOC3: apolipoprotein CIII; F2: thrombin; ENSOARP00000002890: complement C4-like isoform X1; SERPINC1: antithrombin-III precursor; KNG1: kininogen-1 isoform X2.

Supplemental Methods

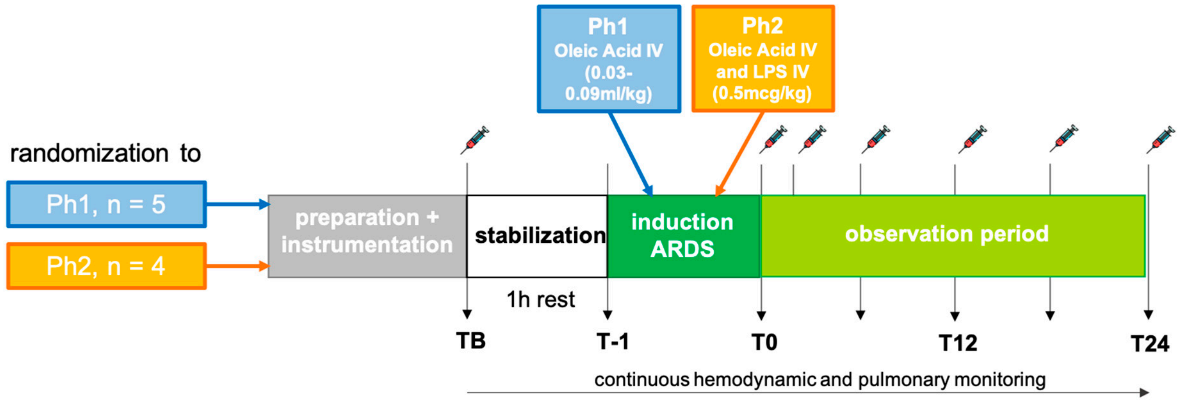

Figure S5 Study design and time line. Abbreviations: ARDS: Acute Respiratory Distress Syndrome; Ph1 and Ph2: phenotype 1 and 2; IV: intravenously; LPS: lipopolysaccharides; T: time point; TB: baseline time point

Adherence to main features of experimental ALI according to the ATS definition

The main features of experimental animal models of ALI are:

a) *Histological evidence of tissue injury* is reported in the LIS score that consists of the components alveolar neutrophils, interstitial neutrophils, hyaline membranes and septal thickening (Figure S2).

b) *Alteration of the Alveolar Capillary Barrier* is reported as the extent of extravascular lung water (Table S1 and S2).

c) *Inflammatory Response* to the lung injury is demonstrated in Figure S1 as cytokine levels at different time points.

d) *Physiological Dysfunction* is described as the extent of decrease in oxygenation and alteration in pulmonary mechanics (Table S1 as compared to Table S2).
